# Supplementary material for: Convalescent Plasma Efficacy in Life-Threatening COVID-19 Patients Admitted to the ICU: A Retrospective Cohort Study
Source: J Clin Med. 2021 May 14;10(10):2113. doi: 10.3390/jcm10102113 (PMC8153619; doi:10.3390/jcm10102113)
Supplement: Supplementary file 1 [file jcm-10-02113-s001.zip › jcm-1178252-supplementary.pdf]

Supplementary material

Table S1. Univariate Cox regression analysis to predict time to clinical improvement.

Variables	Hazard ratio (95% confidence interval)	p-value
**Convalescent plasma therapy, (refer: no)**	0.75 (0.41-1.37)	0.35
**Convalescent plasma with high neutralizing antibody titers ≥ 1:160, (refer: no)**	0.71 (0.32-1.56)	0.40
**Age, year**	0.97 (0.95-0.99)	**0.003**
**Male, n (%)**	1.15 (0.42-3.18)	0.78
**Body mass index, $kg.m^{-2}$**	1.00 (0.95-1.04)	0.91
**SOFA**	0.93 (0.86-0.99)	**0.04**
**SAPS II**	0.97 (0.95-0.99)	**0.005**
**Patients with comorbidities, (refer: no)**	0.79 (0.47-1.31)	0.37
**Comorbidities distribution, (refer: no)**		
Diabetes mellitus	0.84 (0.51-1.39)	0.50
Hypertension	0.73 (0.44-1.23)	0.24
Chronic artery disease	1.04 (0.38-2.85)	0.94
Chronic kidney disease	1.53 (0.55-4.21)	0.41
**Time from symptoms onset to ICU admission, day**	1.01 (0.95-1.08)	0.73
**Time from symptoms onset to CP administration, day**	0.96 (0.91-1.02)	0.22
**Vital signs on ICU admission**		
Temperature (max), °C	1.45 (0.88-2.39)	0.15
Heart rate, $beats.min^{-1}$	1.00 (0.99-1.01)	0.77
Respiratory rate, $breaths.min^{-1}$	1.00 (0.97-1.03)	0.89
**Laboratory data on ICU admission**		
C-reactive protein, $mg.L^{-1}$	1.00 (1.00-1.00)	0.41
Leucocytes count, $\times 10^9.l^{-1}$	0.94 (0.89-0.99)	**0.034**
Lymphocytes count, $\times 10^9.l^{-1}$	0.63 (0.34-1.17)	0.14
Platelet count, $\times 10^9.l^{-1}$	1.00 (1.00-1.00)	0.76
Procalcitonin, $ng.mL^{-1}$	1.00 (0.99-1.01)	0.94
INR	0.60 (0.19-1.93)	0.39
Activated partial thromboplastin time; s	0.99 (0.95-1.02)	0.56
D-dimer, $\mu g.mL^{-1}$	0.78 (0.65-0.93)	**0.006**
Fibrinogen, $g.L^{-1}$	0.98 (0.84-1.13)	0.75
Ferritin, $\mu g.L^{-1}$	1.00 (1.00-1.00)	0.84
Interleukin 6, $ng.L^{-1}$	1.00 (1.00-1.00)	0.18

Alanine aminotransferase, IU.mL^{-1}	1.00 (0.99-1.00)	0.93
Aspartate aminotransferase, IU.mL^{-1}	0.99 (0.99-1.00)	**0.08**
Total bilirubin, µmol.L^{-1}	0.98 (0.96-1.00)	0.13
Creatinine, µmol.L^{-1}	1.00 (1.00-1.00)	0.65
PaO_2/FiO_2 ratio, mmHg	1.004 (1.001-1.007)	**0.008**
Lactate, mmol.L^{-1}	0.49 (0.28-0.87)	**0.01**
**Treatments (refer: no)**		
Invasive mechanical ventilation (reference: HFNO/NIV)	0.31 (0.18-0.51)	**<0.001**
Vasopressor support	0.26 (0.16-0.44)	**<0.001**
Renal replacement therapy	0.30 (0.16-0.57)	**<0.001**
Extracorporeal membrane oxygenation	-	
Tocilizumab	0.89 (0.42-1.88)	0.77
Hydroxychloroquine	1.41 (0.86-2.33)	0.17
Favipiravir	1.14 (0.65-2.01)	0.65
Lopinavir/ritonavir	1.22 (0.71-2.10)	0.46
Methylprednisolone	1.00 (0.61-1.63)	0.99
**WHO scale 5 (refer: WHO scale 4)**	0.69 (0.41-1.14)	0.14

SOFA, Sequential Organ Failure Assessment; SAPS, Simplified Acute Physiology Score; ICU, intensive care unit; PaO_2, arterial oxygen pressure; FiO_2, inspiratory oxygen fraction; INR, international normalized ratio; WHO, World Health Organization; HFNO, high flow nasal oxygen; NIV, non-invasive therapy.

Table S2. Multivariable Cox regression analysis to predict time to clinical improvement.

Variables	Hazard ratio	95% Confidence interval	*P*-value
Convalescent plasma with high neutralizing antibody titers ≥ 1:160 (refer: no)	1.05	0.40-2.72	0.92
Age, year	0.97	0.95-1.00	0.063
Male, (refer: female)	1.10	0.26-4.66	0.89
BMI, kg.m^{-2}	0.99	0.92-1.06	0.72
SOFA score	1.03	0.90-1.18	0.68
Lactate, mmol.L^{-1}	0.73	0.38-1.38	0.33
Leucocytes count, × 10^9.l^{-1}	1.02	0.94-1.10	0.68
PaO_2/FiO_2 ratio, mmHg	1.00	0.99-1.00	0.94
D-dimer, µg.mL^{-1}	1.00	0.74-1.38	0.95
Aspartate aminotransferase, IU.mL^{-1}	0.99	0.98-1.00	0.051
Time from symptoms onset to convalescent plasma infusion, day	0.98	0.90-1.06	0.60
Invasive mechanical ventilation, (refer: HFNO/NIV)	0.59	0.23-1.48	0.26
Vasopressor support (refer: no)	0.45	0.21-0.99	**0.047**
Renal replacement therapy (refer: no)	0.44	0.15-1.26	0.13
Methylprednisolone, (refer: no)	0.99	0.55-1.80	0.98
Tocilizumab, (refer: no)	1.21	0.38-3.78	0.75
Extracorporeal oxygenation membrane, (refer: no)	-	-	-
Comorbidities, (refer: no)	0.79	0.43-1.45	0.44

HFNO, high flow oxygen; NIV, non-invasive ventilation; PaO_2, arterial oxygen pressure; FiO_2, inspiratory oxygen fraction; SOFA, Sequential Organ Failure Assessment. Test of proportional-hazards assumption (Shoenfeld residuals test): p=0.23.

Table S3. Weighted standardized differences.

Variables	Weighted standardized differences (%)
Age, year	14
Male	1
BMI, $kg.m^{-2}$	0.8
SOFA score	5.7
Lactate, $mmol.L^{-1}$	7.8
Leucocytes count, $\times 10^9.l^{-1}$	1.4
PaO_2/FiO_2 ratio, mmHg	18.5
D-dimer, $\mu g.mL^{-1}$	8.8
Aspartate aminotransferase, $IU.mL^{-1}$	10.0
Time from symptoms onset to convalescent plasma infusion, day	15.0
Invasive mechanical ventilation	15.4
Vasopressor support	8.0
Renal replacement therapy	18.9
Methylprednisolone	16.0
Tocilizumab	20
Extracorporeal oxygenation membrane	0.02
Comorbidities	19.5

PaO_2, arterial oxygen pressure; FiO_2, inspiratory oxygen fraction; SOFA, Sequential Organ Failure Assessment.
